# Prognostic Role of Pre-Treatment Metabolic Parameters and Sarcopenia Derived by 2-[^18^F]-FDG PET/CT in Elderly Mantle Cell Lymphoma

**DOI:** 10.3390/jcm11051210

**Published:** 2022-02-23

**Authors:** Domenico Albano, Nadia Pasinetti, Francesco Dondi, Raffaele Giubbini, Alessandra Tucci, Francesco Bertagna

**Affiliations:** 1 Nuclear Medicine, ASST Spedali Civili Brescia, 25128 Brescia, Italy; f.dondi@outlook.com (F.D.); francesco.bertagna@unibs.it (F.B.); 2 Nuclear Medicine Department, University of Brescia, 25121 Brescia, Italy; raffaele.giubbini@unibs.it; 3 Radiation Oncology Department, ASST Valcamonica Esine and University of Brescia, 25128 Brescia, Italy; nadia.pasinetti@unibs.it; 4 Hematology, ASST Spedali Civili of Brescia, 25128 Brescia, Italy; alessandra.tucci@asst-spedalicivili.it

**Keywords:** Mantle Cell Lymphoma, PET/CT, MTV, TLG, sarcopenia, ^18^F-FDG

## Abstract

The goal of this retrospective study was to analyze and compare the prognostic role of fluorine-18-fluorodeoxyglucose positron emission tomography/computed tomography (2-[^18^F]-FDG PET/CT) features and sarcopenia, estimated by CT of PET in elderly (≥65 years) Mantle Cell Lymphoma (MCL). We recruited 53 patients, who underwent pre-treatment 2-[^18^F]-FDG PET/CT and end-of-treatment PET/CT, and the main semiquantitative parameters were calculated. Sarcopenia was measured as skeletal muscle index (SMI, cm^2^/m^2^) and derived by low-dose PET/CT images at the L3 level. Specific cut-offs for SMI were calculated by receiver operator curve and divided by gender. Metabolic response was evaluated at end-of-treatment PET/CT, applying the Deauville score. Progression Free Survival (PFS) and Overall Survival (OS) were calculated for the whole population and for different subgroups, defined as per different sarcopenia cut-off levels. The specific cut-offs to define sarcopenia were 53 cm^2^/m^2^ for male and 45.6 cm^2^/m^2^ for female. Thirty-two (60%) patients were defined as sarcopenic. The 3-year and 5-year PFS rates were 29% and 23%, while the 3-year and 5-year OS rates were 43% and 33%. Metabolic response, total metabolic tumor volume (tMTV), total lesion glycolysis (tTLG) and sarcopenia were independent prognostic factors for PFS. Considering OS, no variable was significantly associated. Combination between PET features and sarcopenia may help to predict PFS.

## 1. Introduction

Mantle Cell Lymphoma (MCL) is a non-Hodgkin’s lymphoma (NHL), with aggressive behavior and poor prognosis representing less than 10% of all NHLs [1,2]. MCL is an NHL frequent in advanced age; the median age at diagnosis ranges from 60 to 70 [3]. The prognosis of elderly lymphoma patients is, of course, worse than that of younger patients; this is probably due to the presence of age-related conditions and comorbidities that could increase the risk of side effects after treatments and worsen the prognosis. Sometimes, the chemotherapy regimen needs to be changed during the course of therapy, with a de-escalation of doses or a reduction in the cycles [4]. The risk of relapse or progression is very high and, nowadays, no clear and shared prognostic factors are available. Furthermore, the MCL International Prognostic Index (MIPI), proposed as the prognostic index, found controversial evidence in the literature [5,6]. The potential prognostic role of fluorine-18-fluorodeoxyglucose positron emission tomography/computed tomography (2-[^18^F]-FDG PET/CT) and its features is yet an open question [7]. Several recent papers [8,9,10] demonstrated that metabolic response after first-line therapy, metabolic tumor volume (MTV) and total lesion glycolysis (TLG) were significantly related with progression free survival (PFS), not with overall survival (OS). A detection of shared prognostic criteria could be fundamental with the aim to stratify patients and personalize the patient management and treatment. In this scenario, the potential prognostic role of sarcopenia and its indexes are not evaluated. Sarcopenia can be considered a multi-factorial syndrome, defined by a progressive and generalized loss of strength and skeletal muscle mass, which may increase the risk of adverse events (until death) and reduce the quality of life [11]. In HL and diffuse large B cell lymphoma (DLBCL), the sarcopenia estimated by CT (low-dose CT of PET or high-dose CT) has been shown to be an independent prognostic factor [12,13], while in MCL, these analyses are anecdotal with low numbers [14,15] of patients included and mixed with other lymphoma histotypes [16,17].

For these reasons, the aim of this retrospective study was to analyze and compare the prognostic value of PET/CT features and sarcopenia, estimated by CT of PET in elderly MCL.

## 2. Materials and Methods

### 2.1. Patients

**Figure 1:**
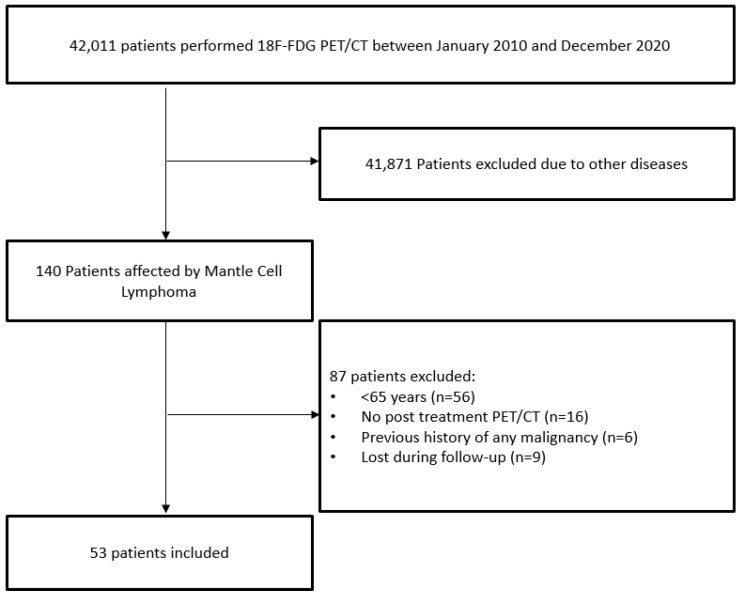
Flowchart of patients included.

We have retrospectively screened about 42.011 2-[^18^F]-FDG PET/CT scans performed in our Nuclear Medicine Department from January 2010 until December 2020 for any reason. Among them, 140 had a histological diagnosis of MCL. The other patients had different oncological or not oncological diseases. These patients were further selected according to some inclusion criteria: (1) patients who performed a baseline PET/CT scan and an end-of-treatment (eot) PET/CT after first line chemotherapy; (2) patients without a previous history of other malignancies; (3) patients with an age at the time of MCL diagnosis ≥65 years; (4) patients with at least 12 months of follow-up from the baseline 2-[^18^F]-FDG PET/CT. Finally, 53 patients were recruited (Figure 1). The medical records of these patients were analyzed with attention to the main epidemiological (gender, age at diagnosis, BMI), clinical (B symptoms, MIPI score, β2-microglobulin level and lactate-dehydrogenase (LDH) level), histopathological (blastoid variant), size (bulky disease, splenomegaly), PET/CT semiquantitative data and sarcopenia features. MIPI score, β2-microglobulin and LDH values were dichotomized using a cutoff value of 2, 2.8 mg/L and 245 U/L respectively.

All patients were treated according to the institution’s standard protocol with chemotherapy regimen. In 22 cases R-BAC (Rituximab, Bendamustine and Cytarabine) regimen was performed up to six cycles of immuno-chemotherapy and in three cases, up to four cycles; in ten cases R-CHOP (Rituximab, Cyclophosphamide, Hydroxydoxorubicine, Oncovin and Prednisone) regimen was done up to six cycles and in five, up to three cycles; in three cases R-hyper-CVAD (rituximab, hyper-fractionated cyclophosphamide, vincristine, adriamycin, and dexamethasone alternating with rituximab, high-dose methotrexate, and high-dose cytarabine) regimen was chosen and in the remaining ten patients MCL 0208 trial which consisted of high-dose chemotherapy plus Rituximab, followed by Lenalidomide and autologous stem cell transplantation as maintenance therapy was used.

There was a minimal overlap (n 11 patients) with a previous paper already published [9].

### 2.2. 2-[^18^F]-FDG PET/CT Imaging and Interpretation

All patients underwent a baseline 2-[^18^F]-FDG PET/CT scan before any kind of therapy and, if available, an eotPET/CT. Then, 2-[^18^F]-FDG PET/CT was acquired after at least 4 h fasting and with glucose blood level <150 mg/dL. An activity of 3.5–4.5 MBq/Kg of radiotracer was injected intravenously and scans began about 60 ± 10 min after the injection. The scan was performed from the skull base to the mid-thigh on two PET/CT scanners: Discovery ST and Discovery 690 PET/CT tomographs (General Electric Company—GE^®^—Milwaukee, WI, USA) with standard parameters (CT: 80 mA, 120 kV without contrast; 2.5–4 min per bed-PET-step of 15 cm). The matrix of reconstruction was 256 × 256 and the field of view was 60 cm. For both tomographs a standard non-contrast free-breathing helical low-dose CT was obtained for morphologic correlation and attenuation correction. The D-STE acquisition parameters were: 120 kV, fixed tube current ~73 mAs (40–160 mAs), 4 slices × 3.75 mm and 3.27 mm interval, pitch 1.5:1, tube rotation 0.8 s. The D690 acquisition parameters were: 120 kV, fixed tube current ~60 mAs (40–100 mAs), 64 slices × 3.75 mm and 3.27 mm interval, pitch 0.984:1, tube rotation 0.5 s. For D690, time-of-flight (TOF) and point spread function (PSF) were used as reconstruction algorithms; filter cutoff 5 mm, 18 subsets; three iterations. For D-STE, ordered subset expectation maximization (OSEM) was applied; filter cutoff 5 mm; 21 subsets, two iterations.

When available, eotPET/CT were performed at least three weeks after the last cycle of chemotherapy.

All PET images were analyzed visually and semi-quantitatively by a nuclear medicine physician with experience (DA) with the measurements of the maximum standardized uptake value body weight (SUVbw), the SUVmax corrected for the lean body mass (SUVlbm), the SUVmax corrected for body surface area (SUVbsa), lesion to liver SUVmax ratio (L-L SUV R), lesion to blood-pool SUVmax ratio (L-BP SUV R), MTV and TLG. Eot PET/CT was interpreted visually by the same nuclear medicine physician with more than 10 years of experience (DA) applying the Deauville scores. According to Deauville criteria [18], 2-[^18^F]-FDG PET/CT was interpreted as follows: 1 = no uptake above background, 2 = uptake equal to or lower than mediastinum, 3 = uptake higher than mediastinum and lower than liver, 4 = uptake moderately increased compared to the liver and 5 = uptake markedly increased compared to the liver. After therapy, patients with a score of 1–3 were judged as having complete metabolic response, while patients with score 4–5 as not complete metabolic response. For the measurements of SUV, a region of interest (ROI) was drawn over the area of maximum activity of the lesion with highest uptake and the SUVmax was derived as the highest SUV of the pixels within the ROI. SUVmax of the liver was calculated at the VIII hepatic segment of axial PET images using a round-shape ROI of 10 mm; SUVmax of the blood-pool was calculated at the aortic arch by use not involving the vessel wall in a similar way. MTV was measured from attenuation-corrected PET images using an SUV-based automated contouring program (Advantage Workstation 4.6, GE HealthCare) with an isocounter threshold method based on 41% of the SUVmax, as previously recommended by European Association of Nuclear Medicine because of its high inter-observer reproducibility [19]. Total MTV (tMTV) was obtained by the sum of all nodal and extranodal lesions. Bone marrow involvement was included in volume measurement only if there was focal uptake; splenic involvement was considered if there was focal uptake in spleen or diffuse uptake higher than 150% in the liver background. Total TLG (tTLG) was calculated as the sum of the product of MTV of each lesion and its SUVmean. Semiquantitative analyses were performed by the same nuclear medicine physicians with long experience in lymphoma and in the use of Advantage Workstation 4.6, GE HealthCare for contouring.

### 2.3. Sarcopenia Analysis

Low-dose CT of 2-[^18^F]-FDG PET/CT scans were analyzed by a researcher for the estimation of muscular and adipose tissues using Slice-O-Matic software V4.2 (Montreal, QC, Canada Tomovision). A transaxial slice with a multiplanar reconstruction at the third lumbar (L3) level was considered for the measurement of skeletal muscle area (SMA) considering psoas, paraspinal, abdominal transverse rectum, internal and external oblique, because the skeletal muscle in this area has been shown to represent the whole-body tissue quantities [11]. To measure the SMA (cm^2^), CT Hounsfield unit thresholds were –29 to 150. If necessary, the tissue margins were manually corrected. Subsequently SMA was normalized for the height to obtain the skeletal muscle index (SMI) expressed in cm^2^/m^2^.

### 2.4. Statistical Analysis

MedCalc (Belgium) was used as software for statistical analysis. Categorical variables were expressed as simple and relative frequencies; the numeric variables were expressed as mean, standard deviation, minimum and maximum.

The receiver operating characteristic (ROC) curve analysis was carried out to find the best threshold point for each metabolic variable and SMI, which predict the risk of progression/relapse (Supplementary Table S1). Progression free survival (PFS) was calculated from the date of pre-treatment 2-[^18^F]-FDG PET/CT to the date of first disease progression, relapse, death or the date of last follow-up. Overall survival (OS) was calculated from the date of pre-treatment 2-[^18^F]-FDG PET/CT to the date of death from any cause or to the date of last follow-up. Survival curves were plotted according to the Kaplan–Meier method and differences between groups were analyzed by using a two-tailed log rank test. Cox regression was used to estimate the hazard ratio (HR) and its confidence interval (CI). A *p* value of < 0.05 was considered statistically significant.

## 3. Results

### 3.1. Patients Features

Among 53 patients included, there was a prevalence of male patients (74%); the average age was 72.7 years old. Most patients had advanced tumor stage disease, with stage IV in 45 patients. B-symptoms were present in 10 patients, while bulky disease in 6 cases. LDL and β2 microglobulin were higher than normal range values in 19 and 18 patients, respectively. Table 1 summarizes the clinical characteristics of the population. Pathological increased ^18^F-FDG-uptake was present in all patients, showing the presence of at least one nodal or extranodal hypermetabolic lesion. The average SUVbw was 9.6 (range 3.3–27), average SUVlbm was 7.4 (range 2.4–22.3), average SUVbsa was 2.4 (range 0.9–7.3), average L-L SUV R 46.27 (1.7–54), average L-BP SUV R 5.3 (1–44), average tMTV was 358 cm^3^ (3–1800 cm^3^) and average TLG was 2023 (10–20088). The average SMI was 49.9 cm^2^/m^2^ (range 36.3–67) and was significantly higher in men, with a mean of 53.1 cm^2^/m^2^ (range 39–67), than women, with a mean value of 42 cm^2^/m^2^ (range 36.3–46.3) (Table 1). Based on Lugano classification metabolic response [18], 30 (57%) patients had a complete metabolic response at eotPET/CT. Fifteen (28%) patients had partial metabolic response and five (9%) patients had progression of disease. Three patients died before the execution of eotPET/CT.

### 3.2. Sarcopenic Analysis

For the definition of sarcopenia, in the absence of specific shared thresholds based on the MCL population, we derived our thresholds by applying ROC curve analysis. A separate analysis for male and female was performed (Supplemental Table S1). The gender-specific cut-offs were 53 cm^2^/m^2^ for male and 45.6 cm^2^/m^2^ for female. With these cut-offs, 32 (60%) of our patients were considered sarcopenic. There was a significantly higher prevalence of sarcopenia in females than males (93% vs. 47%, *p* = 0.001), with no significant differences found considering age, BMI, tumor stage, B symptoms, bulky disease, blood samples (LDH, β 2 microglobulin), MIPI score and complete metabolic response at eot PET (Table 2). Instead, the presence of the blastoid variant was significantly higher in patients with sarcopenia. Focusing on the relationship between semiquantitative PET/CT variables and sarcopenia, no significant differences among SUVbw, SUVlbm, SUVbsa, lesion to BP SUVmax ratio and lesion to liver SUVmax ratio were registered, comparing sarcopenic and not sarcopenic cases. Further, tMTV and tTLG were significantly higher in patients with low SMI.

### 3.3. Survival Analysis

**Figure 2:**
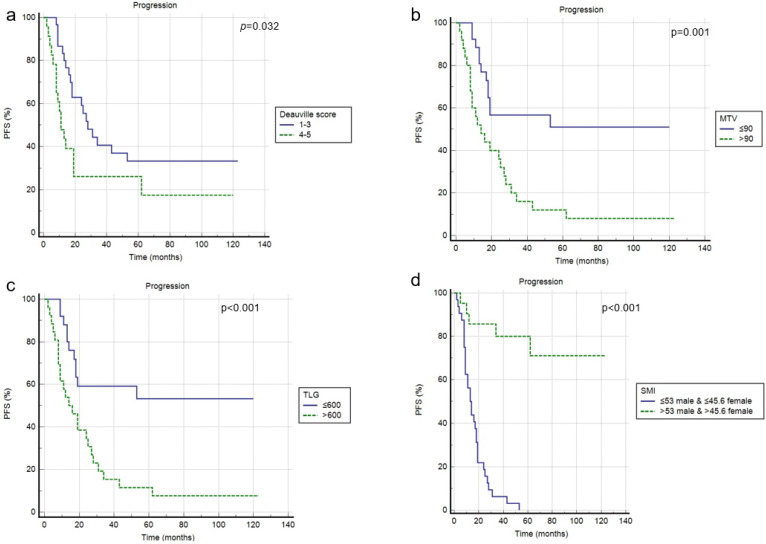
Progression free survival according to Deauville score (**a**), tMTV (**b**), tTLG (**c**) and SMI (**d**).

**Figure 3:**
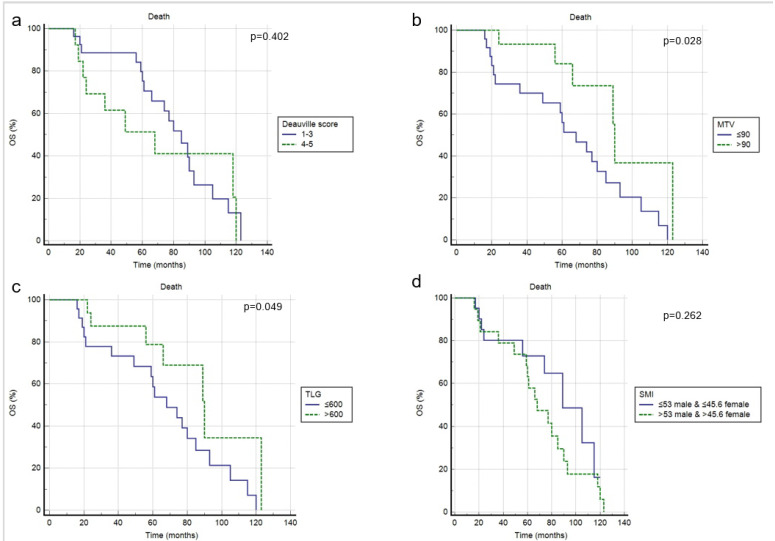
Overall survival according to Deauville score (**a**), tMTV (**b**), tTLG (**c**) and SMI (**d**).

**Figure 4:**
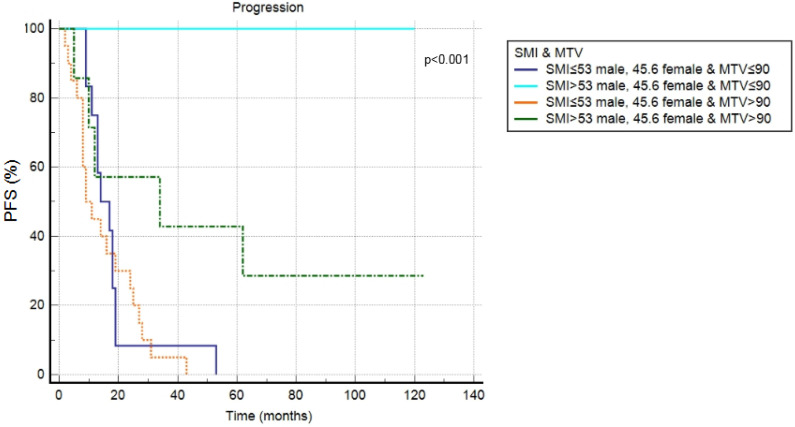
Kaplan–Meier curve considering the combination of tMTV and SMI.

At a median follow-up of 50 months, a progression of disease or relapse was registered in 37 patients (70%), with an average time of 17.2 months (range: 2–62 months), while death occurred in 26 patients (49%), with a mean time of 33.6 months (range: 2–120). Overall, the 3-year and 5-year PFS rates were 29% and 23%, while the 3-year and 5-year OS rates were 43% and 33%. In univariate analysis, blastoid variant, Deauville Score 4–5, tMTV, tTLG and SMI were significantly correlated with PFS (*p =* 0.015, *p =* 0.032, *p =* 0.001, *p* < 0.001, *p* < 0.001), while the other clinical and metabolic features were not (Figure 2, Table 3). In multivariate analysis, the Deauville Score 4–5, tMTV, tTLG and SMI were confirmed to be independent prognostic factors. Considering OS, only tMTV and tTLG were shown to be significantly related to outcome at univariate analysis, but not at multivariate analysis. SMI demonstrated no prognostic impact (Figure 3, Table 3). Combining tMTV and SMI thresholds, patients without sarcopenia and with low metabolic volume disease (<90 cm^3^) had the best PFS, without any case of progression/relapse during the follow-up. The worst PFS was specific of patients with sarcopenia independent from the tMTV value (Figure 4).

## 4. Discussion

The clinical meaning of the sarcopenia has already been investigated and it is emerging as a promising tool for predicting the survival and treatment response [20,21,22,23]. However, in lymphoma patients, its usefulness is not yet clear because controversial evidence is available and the studies present are based specifically on HL and DLBCL [12,24], while for the other lymphoma histotyopes, specific studies are lacking. For this reason, we aimed to analyze patients affected by MCL, which is a lymphoma typical in advanced age. Just in this setting, the evaluation of the role of sarcopenia may be fundamental. For the measurements of sarcopenia, several techniques and imaging examinations have been proposed, such as hand-held dynamometer, CT and MRI [25,26]. These tools are very different for availability, cost, ease of execution and accuracy. CT seems to have the best compromise and it is often applied for the evaluation of the muscular area, due to its ability to distinguish adipose and muscular tissue from other soft tissues of the body. A recent paper demonstrated a good agreement and reproducibility between low-dose CT of PET and high-dose CT in the measurements of the adipose and muscular area [27]. These results underlined the possibility to use 2-[^18^F]-FDG PET/CT, both for the measurements of “classical” PET/CT parameters (like SUV, MTV, TLG) and sarcopenia.

To the best of our knowledge, no studies about the rate and the role of sarcopenia in MCL are available, particularly in advanced age patients. We decided to select elderly MCL patients, because they are, by definition, frail patients with a high risk of toxicities and poor prognosis.

Due to the lack of consensus for optimal cut-off levels to define sarcopenia in lymphoma patients, and especially in MCL, we derived specific values for our population using ROC curve analysis. Many different thresholds have been proposed [12], specific for gender, BMI, ethnicity and geographic regions. Thus, it is hazardous to consider a unique threshold validated in other lymphoma variants. The cut-off values obtained in our analysis were 53 cm^2^/m^2^ for males and 45.6 cm^2^/m^2^ for females. Further studies with larger samples are essential to confirm or change these results, especially for women, where our cases are relatively low (n 14). However, these values may be considered a starting point to investigate. With these cut-offs, we found a prevalence of sarcopenia in 60% of our sample and it was more diffused in women than men. Besides gender, only the blastoid variant is significantly related to sarcopenia status, assuming a more aggressive disease in blastoid CML [28].

The second goal of our study was to analyze the prognostic role of several variables, including 2-[^18^F]-FDG PET/CT features and sarcopenia, in terms of PFS and OS.

Besides quantitative parameters, visual analysis, applying the Deauville score, remains a valid prognostic variable for PFS in MCL, as demonstrated by several papers and confirmed in our analysis [9,29].

Among 2-[^18^F]-FDG PET/CT parameters, MTV and TLG were shown to be superior than SUV-related factors, but also in this case, the significant correlation from the multivariate analysis was confirmed only for PFS. The same evidence was revealed considering sarcopenia. However, the combination of tMTV and sarcopenia better predicts the PFS. The absence of prognostic factors to predict OS may be explained by the advanced age of patients, which probably significantly influenced the outcome; moreover, it could be other variables not evaluated in this study with a prognostic impact. The open question, not yet resolute, is the choice of the cut-off values to apply to define sarcopenia with CT.

Several limitations affect the quality of this work, such as the retrospective study design, the heterogeneous management received by patients (for example primary treatment) and the relatively low number of patients included due to the inclusion criteria chosen. This is the first attempt to investigate the prognostic role of sarcopenia, detected by CT in combination with PET/CT features, in elderly MCL. Prospective studies are warranted to better define, in real-life settings, whether these easy and patient-level approaches retain their significance and utility and could be used to improve treatment tailoring in the setting of elderly MCL.

## 5. Conclusions

Baseline evaluation CT of PET may help to define sarcopenia, with a specific cutoff for gender in elderly MCL. Sarcopenia, together with Deauville score, tMTV and tTLG, may help to predict PFS. Instead, they had no role in predicting OS.

## Figures and Tables

**Table 1:** Main features of our sample (53 patients).

	Patients *n* (%)	Average ± SD (Range)
Age (years)		72.7 ± 5.6 (66–88)
Sex male	39 (74%)	
Sex female	14 (26%)	
BMI		26.15 ± 4.4 (16.8–34.8)
Tumor stage at diagnosis (Ann Arbor)		
I	0 (0%)	
II	4 (7.5%)	
III	4 (7.5%)	
IV	45 (85%)	
B symptoms	10 (19%)	
Blastoid variant	7 (13%)	
Bulky disease	6 (11%)	
LDH ≤ 245	34 (64%)	
>245	19 (36%)	
β 2 microglobulin ≤ 2.8	35 (66%)	
>2.8	18 (34%)	
Ki-67 score ≤ 15%	22 (44%)	
>15%	28 (56%)	
MIPI score ≤ 2	12 (23%)	
(>2)	41 (77%)	
SUVbw		9.6 ± 5.3 (3.3–27)
SUVlbm		7.4 ± 4.4 (2.4–22.3)
SUVbsa		2.4 ± 1.4 (0.9–7.3)
Lesion to BP SUVmax ratio		6.2 ± 8.8 (1.7–54)
Lesion to liver SUVmax ratio		5.3 ± 6.8 (1–44)
tMTV		358 ± 481 (3–1800)
tTLG		2023 ± 3147 (10–20,088)
SMI		49.9 ± 7.7 (36.3–67)
for male		53.1 ± 6.3 (39–67)
for female		42 ± 3.4 (36.3–46.3)

BMI: body mass index; LDH: lactate dehydrogenase; MIPI: Mantle international prognostic index; SUV: standardized uptake value; bw:body weight; lbm: lean body mass; bsa: body surface area; BP: blood pool; MTV: metabolic tumor volume; TLG: total lesion glycolysis; SMI: skeletal muscle index; SD: standard deviation.

**Table 2:** Comparison of baseline variables between sarcopenic and not sarcopenic patients applying cut-offs of 53 cm^2^/m^2^ for male and 45.6 cm^2^/m^2^ for female.

	Sarcopenia *n* = 32	Not Sarcopenia *n* = 21	*p* Value
Male:Female	21:13	18:1	0.001
Age (mean ± SD)	73.4 ± 5.7	71.4 ± 5.2	0.338
BMI	25.8	26.6	0.801
Tumor stage advanced	29 (91%)	19 (90%)	0.985
Bulky disease	4 (12.5%)	2 (10%)	0.745
Splenomegaly	14 (44%)	6 (29%)	0.093
Blastoid variant	7 (22%)	0 (0%)	0.021
LDH (mean ± SD)	276 ± 264	208 ± 83	0.257
β 2 microglobulin (mean ± SD)	4.5 ± 5.5	2.44 ± 2	0.526
MIPI score > 2	10 (31%)	5 (24%)	0.288
Complete metabolic response	17 (57%)	13 (62%)	0.537
SUVbw (mean ± SD)	8.7 ± 4.9	10.8 ± 5.7	0.139
SUVlbm (mean ± SD)	6.7 ± 4	8.35 ± 4.8	0.144
SUVbsa (mean ± SD)	2.2 ± 1.3	2.7 ± 1.6	0.160
Lesion to BP SUVmax ratio (mean ± SD)	4.7 ± 5.7	6.3 ± 10	0.411
Lesion to liver SUVmax ratio (mean ± SD)	5.4 ± 4.8	7.4 ± 9	0.402
tMTV (mean ± SD)	470 ± 57	192 ± 34	0.040
tTLG (mean ± SD)	2726 ± 378	985 ± 138	0.033

BMI: body mass index; LDH: lactate dehydrogenase; MIPI: mantle international prognostic index; SUV: standardized uptake value; bw: body weight; lbm: lean body mass; bsa: body surface area; BP: blood pool; MTV: metabolic tumor volume; TLG: total lesion glycolysis; SD: standard deviation.

**Table 3:** Univariate and multivariate analyses for PFS and OS.

	Univariate Analysis		Multivariate Analysis	
	*p* Value	HR (95% CI)	*p* Value	HR (95% CI)
**PFS**				
Sex	0.299	1.358 (0.500–2.333)		
Age	0.298	1.225 (0.284–2.585)		
MIPI score	0.582	1.989 (0.500–4.212)		
LDH level	0.121	0.610 (0.292–1.154)		
Β2 microglobulin	0.895	1.053 (0.476–2.333)		
Bulky disease	0.458	0.124 (0.102–1.715)		
Splenomegaly	0.248	1.582 (0.123–3.002)		
Blastoid variant	0.015	1.292 (1.101–1.500)	0.102	1.250 (0.888–1.650)
Deauville score	0.032	2.155 (1.068–4.351)	0.042	2.255 (1.250–3.690)
SUVbw *	0.555	1.446 (0.759–3.042)		
SUVlbm *	0.434	1.111 (0.534–2.126)		
SUVbsa *	0.331	1.459 (0.339–2.856)		
L-L SUV R *	0.450	1.107 (0.756–2.122)		
L-BP SUV R *	0.324	1.222 (0.444–4.235)		
tMTV *	0.001	3.190 (1.568–6.374)	0.039	2.833 (1.053–7.619)
tTLG *	<0.001	3.258 (1.638–6.479)	0.022	2.075 (0.889–4.843)
SMI *	<0.001	0.125 (0.062–0.253)	<0.001	0.031 (0.007–0.132)
R-BAC vs other	0.401	0.852 (0.222–1.589)		
**OS**				
Sex	0.211	1.666 (0.389–5.026)		
Age	0.375	1.389 (0.445–3.005)		
MIPI score	0.690	0.987 (0.666–1.589)		
LDH level	0.480	0.825 (0.450–1.454)		
Β2 microglobulin	0.333	0.858 (0.420–1.689)		
Bulky disease	0.387	1.258 (0.555–2.297)		
Splenomegaly	0.222	1.359 (0.801–1.987)		
Blastoid variant	0.342	0.659 (0.350–1.259)		
Deauville score	0.402	1.404 (0.596–3.310)		
SUVbw *	0.453	0.816 (0.464–1.408)		
SUVlbm *	0.160	0.698 (0.368–1.179)		
SUVbsa *	0.207	0.677 (0.363–1.245)		
L-L SUV R *	0.125	0.689 (0.376–1.127)		
L-BP SUV R *	0.307	0.631 (0.306–1.452)		
tMTV *	0.028	0.374 (0.172–0.811)	0.129	0.563 (0.105–1.009)
tTLG *	0.049	0.448 (0.207–0.970)	0.062	0.550 (0.102–1.120)
SMI *	0.262	1.574 (0.736–3.365)		
R-BAC vs. other	0.396	0.701 (0.386–1.499)		

PFS: progression free survival; OS: overall survival; HR: hazard ratio; CI: confidence interval; N°: number; SUV: standard uptake value; bw: body wheight; lbm: lean body mass; bsa: body surface area; L-L R: lesion to liver ratio; L-BP R: lesion to blood pool ratio; MTV: total metabolic tumor volume; TLG: total lesion glycolysis. * Variables dichotomized using cutoff values after ROC analysis reported in Supplemental Table S1.

## Data Availability

Data are not public, but are present in our institution.

## Supplementary Materials

The following supporting information can be downloaded at: https://www.mdpi.com/article/10.3390/jcm11051210/s1, Supplemental Table S1: Receiver operating characteristic (ROC) curve analysis of metabolic and sarcopenic PET/CT parameters.
