# Supplementary material for: Prognostic Role of Pre-Treatment Metabolic Parameters and Sarcopenia Derived by 2-[18F]-FDG PET/CT in Elderly Mantle Cell Lymphoma
Source: J Clin Med. 2022 Feb 23;11(5):1210. doi: 10.3390/jcm11051210 (PMC8911178; doi:10.3390/jcm11051210)
Supplement: Supplementary file 1 [file jcm-11-01210-s001.zip › jcm-1550632-supplementary.pdf]

Supplemental Table S1: Receiver operating characteristic (ROC) curve analysis of metabolic and sarcopenic PET/CT parameters

	ROC curve				
**Parameter**	cutoff	AUC (95% CI)	p value	Sens (95% CI)	Spec (95% CI)
SUVbw	9.5	0.578 (0.433-0.714)	0.371	69% (52-84)	50% (25-75)
SUVlbm	7.2	0.555 (410-693)	0.534	67% (49-81)	50% (25-75)
SUVbsa	2.2	0.556 (0.412-0.694)	0.531	67% (49-81)	50% (25-75)
L-L SUV R	3.68	0.556 (0.411-0.693)	0.526	56% (38-72)	62.5% (35-85)
L-BP SUV R	8.47	0.527 (0.384-0.667)	0.766	97% (85.5-100)	19% (4-46)
tMTV	90	0.800 (0.666-0.898)	<0.001	67% (49.-81)	87.5% (62-98)
tTLG	600	0.778 (0.641-0.881)	<0.001	67% (49.-81)	87.5% (62-98)
SMI male	53	0.869 (0.722-0.955)	<0.001	80% (59-93)	100% (77-100)
SMI female	45.6	1.000 (0.768-1.000)	<0.001	100% (73.5-100)	100% (16-100)

AUC: area under curve; *CI*: confidence interval; *sens:* sensibility; *spec:* specificity; *SUV:* standardized uptake value; *bw*:body weight; *lbm:* lean body mass; *bsa:* body surface area; *L-L R:* lesion to liver ratio; *L-BP R:* lesion to blood pool ratio; *MTV:* total metabolic tumor volume; *TLG:* total lesion glycolysis; *SMI*: skeletal muscle index.
